# Arrestin Domain Containing 3 Reverses Epithelial to Mesenchymal Transition and Chemo-Resistance of TNBC Cells by Up-Regulating Expression of miR-200b

**DOI:** 10.3390/cells8070692

**Published:** 2019-07-10

**Authors:** Young Hwa Soung, Heesung Chung, Cecilia Yan, Jingfang Ju, Jun Chung

**Affiliations:** 1 Department of Pathology, Stony Brook Medicine, Stony Brook, New York, NY 11794, USA; 2 Department of Life Sciences, Ewha Womans University, Seoul 03760, Korea

**Keywords:** ARRDC3, miR-200b, 5-fluorourancil (5-FU), epithelial to mesenchymal transition (EMT), chemo-resistance, triple negative breast cancer (TNBC)

## Abstract

Our previous studies demonstrated the importance of arrestin domain containing 3 (ARRDC3), a metastasis suppressor, in inhibiting invasive and metastatic potential of triple negative breast cancer (TNBC) in vitro and in vivo. However, little is known about ARRDC3 mediated transcriptional control and its target genes that are implicated in its metastatic suppressing activity. In this study, we used miRNA array and subsequent functional analyses to identify miRNAs whose expression are significantly regulated by ARRDC3 in TNBC cells. We identified miR-200b as a major target gene of ARRDC3. miR-200b played an essential role in mediating ARRDC3 dependent reversal of EMT phenotypes and chemo-resistance to DNA damaging agents in TNBC cells. Expression of miR-200b also increased the expression of ARRDC3 as well in TNBC cells, suggesting a positive feedback loop between these two molecules. In addition, we combined the therapeutic powers of miR-200b and 5-fluorourancil (5-FU) into a single compound (5-FU-miR-200b) to maximize the synergistic effects of these compounds. Chemically modified miR-200b (5-FU-miR-200b mimic) was more effective in inhibiting metastatic potentials of TNBC cells than unmodified miR-200b and does not require transfection reagents, implying its therapeutic potential in TNBC. Our studies showed the importance of therapeutic targeting ARRDC3/miR-200b pathway in TNBC.

## 1. Introduction

Breast cancer is the most common type of cancer in women globally [1]. Although significant advances in the treatment and detection of breast cancer have been made in recent years, the survival rate of patients with metastatic breast cancer has been dropping [1,2]. Accounting for approximately 15–20% of all total breast cancer cases, triple negative breast cancer (TNBC) is one of most aggressive breast cancer subtypes, with poorer prognosis than ER/PR positive or Her2 overexpressed breast cancer due to a lack of targeted therapy [2,3,4]. The treatment of TNBC patients is limited to cytotoxic drugs such as 5-fluorourancil (5-FU), paclitaxel, doxorubicin, but chemoresistance is the major reason for failure of chemotherapy and mortality in TNBC [5,6,7]. Further, the molecular mechanism(s) involved in chemoresistance and strategies to resensitize chemoresistant cancer cells to chemotherapy remain to be determined in TNBC.

We previously demonstrated that expression of arrestin domain containing 3 (ARRDC3), a potential metastatic suppressor, is suppressed in metastatic TNBC cells due to epigenetic silencing [8]. Our subsequent study showed that either a forced expression of ARRDC3 or treatment of small molecule compounds such as selinexor that restore the levels of ARRDC3 effectively inhibit TNBC functions important for progression in vitro and in vivo [9]. Metastasis and tumor-suppressing roles of ARRDC3 in multiple cancer models including breast cancer have been demonstrated recently [9,10,11]. One of the mechanisms by which ARRDC3 contributes to metastasis suppressing functions is through inducing ubiquitination and degradation of phosphorylated β2-adrenergic receptor (β2 AR) and integrin β4 (ITG β4) by serving as an adaptor molecule between these receptors and E3 ligase [10,11]. Despite the importance of ARRDC3 as a tumor and metastasis suppressor, little is known about ARRDC3 mediated transcriptional control and its target genes that contribute to its metastatic and chemo-resistance suppressing activity of ARRDC3.

In the current studies, we studied the mechanism by which ARRDC3 sensitizes TNBC cells to DNA damaging chemotherapeutic agents such as 5-FU that is not currently known as a successful TNBC treatment option. As epigenetic changes including miRNA expression has been implicated in aggressive nature of cancers [12,13,14], we explored the relationship between ARRDC3 and specific miRNAs important in controlling invasive and chemo-resistant potentials of TNBC. miRNA array based screening and subsequent functional analysis of target miRNAs whose expression are regulated by ARRDC3 expression in TNBC cells led to the finding that miR-200b-3p (miR-200b) is an important target gene of ARRDC3 in mediating its metastatic suppressing and chemo-sensitizing functions. While the role of miR-200b in chemo-sensitization and tumor suppressing functions in hepatocellular carcinoma and glioma models has been demonstrated [15,16,17], therapeutic strategy involving miR-200b in TNBC models has not been developed yet. Our studies here addressed the mechanism by which ARRDC3 sensitizes TNBC cells to DNA damaging agents such as 5-FU via miR-200b and developed the novel therapeutic strategy that integrates the therapeutic powers of 5-FU and miR-200b into a single compound for the potential novel treatment option for TNBC.

## 2. Materials and Methods

### 2.1. Cell Lines and Reagents

MCF7, MDA-MB-468 and MDA-MB-231 breast cancer cells were maintained in DMEM with 1 g/L glucose, L-glutamine and sodium pyruvate formulation, supplemented with 10% FBS and 1% penicillin/streptomycin. HCC-1937, BT474 and BT549 breast cancer cells were cultured in RPMI-1640 supplemented with 10% FBS and 1% penicillin/streptomycin. All cell lines were purchased from ATCC (Manassas, VA, USA). They were cultured in humidified incubators at 37 °C in 5% CO_2_. 3xHA-ARRDC3 and GFP-ARRDC3 vectors were purchased from the GeneCopoeia, Inc. (Rockville, MD, USA). Transfection of the vector was performed using Lipofectamine LTX-Plus or Lipofectamine 3000 (Thermo Fisher, Waltham, MA, USA) according to the manufacturer’s instruction. Vimentin (D21H3), E-cadherin (24E10), ZO-1 (D7D12), TCF8/ZEB1 (D80D3), Bcl-2 (D55G8) and HA-Tag (C29F4) antibodies were purchased from Cell Signaling (Danvers, MA, USA). ARRDC3 (ab68417) and fibronectin (ab2413) antibodies were obtained from Abcam (Cambridge, MA, USA). β-Actin (clone C-11) antibody was purchased from Santa Cruz Biotechnology (Santa Cruz, CA, USA). Thymidylate Synthase (clone TS106) antibody was purchased from EMD Millipore (Billerica, MA, USA). Selinexor was provided by Karyopharm Therapeutics, Inc (Natick, MA, USA). 5-FU, Cisplatin and paclitaxel were obtained from Selleckchem (Houston, TX, USA).

### 2.2. Western Blot Analysis

Cells were lysed in cold RIPA-EDTA buffer [50 mM Tris, pH 7.4; 150 mM NaCl; 1% NP-40; 0.5% sodium deoxycholate; 0.1% SDS; and 5 mM EDTA] containing 1 mM phenylmethylsulfonyl fluoride, 1 mM Na_3_VO_4_, and protease inhibitor (Thermo Scientific Pierce, Rockford, IL, USA). The protein concentrations were determined using the BCA protein assay kit (Thermo Scientific Pierce). The samples were separated on 4% to 20% gradient SDS PAGE and transferred to PVDF membranes by using the Trans-Blot Turbo transfer system (Bio-Rad, Hercules, CA, USA). The blots were incubated with primary antibodies in TBS-T or TBS-T with 5% *w*/*v* nonfat dry milk, then with appropriate secondary antibodies conjugated to IgG-horseradish peroxidase. Proteins were detected using the Clarity Western ECL blotting substrate (Bio-Rad). All bands were imaged with ChemiDoc Touch Imaging System (Bio-Rad).

### 2.3. miRNA Profiling

miRNA profiling was performed in the laboratory of Ocean Ridge Biosciences (Deerfield Beach, FL, USA). Briefly, RNAs were isolated from GFP and GFP-ARRDC3 overexpressing MDA-MB-231 cells using TRI Reagent^®^ (Molecular Research Center, Cincinnati, OH, USA). 100 nanograms (ng) of low molecular weight RNAs for each sample were 3′-end labeled with Oyster-550 fluorescent dye using the Flash Tag RNA labeling Kit (Genisphere; Hatfield, PA, USA). The labeled RNA samples were hybridized to M19 microRNA microarrays containing 601 human miRNA probes in triplicate. The microarrays were scanned on an Axon Genepix 4000B scanner (Molecular Devices San Jose, CA, USA), and data was extracted from images using GenePix V4.1 software. Statistical comparisons were performed with One-way ANOVA.

### 2.4. miRNA Preparation and Transfection

Pre-miR precursor miR-200b-3p and Pre-miR negative control precursor (NC #2, miR-NC) were purchased from Ambion (Invitrogen). miRIDIAN miRNA-200b-3p mimics were purchased from Dharmacon (Lafayette, CO, USA). To generate chemically modified 5-FU-miR-200b mimics, single-strand RNAs modified with 5-fluorouridine in place of internal U were synthesized in Dharmacon. The 2-ACE protecting groups of RNA oligonucleotides were removed by deprotection reaction according to the manufacturer’s protocol. The equimolar amounts of sense and antisense strand were mixed and annealed to form RNA duplex (called mimics). For miRNA transfection, cells were plated into 6-well plate one day before transfection. 100 nM miRNAs were transfected into MDA-MB-231 cells by using Oligofectamine (Invitrogen-Life Technologies, Carlsbad, CA, USA), according to the manufacturer’s instructions. For oligofectamine free transfection, 100 nM miRNAs were diluted in Opti-MEM and added to cells.

### 2.5. Real-Time qRT-PCR of miR-200b Expression

miR-200b specific primer and the internal control RNU44 and RNU48 gene were purchased from Ambion (Thermo Scientific). cDNA synthesis was performed by High-Capacity cDNA synthesis kit (Applied Biosystems, Foster City, CA, USA), according to manufacturer’s protocol.

Real-time qRT-PCR was carried out using TaqMan Gene Expression Assay (Applied Biosystems) for miR-200b primer on an Applied Biosystems 7500 Real-Time PCR machine. Fold change in expression was determined using the ddCT method after normalizing to control gene.

### 2.6. Identification of miR-200b Target Genes

The miRDB database (http://mirdb.org/miRDB) was used to predict target genes for miR-200b. Putative target genes with the probability of interaction (according to miRDB) given as a target score >90 were selected. Additionally, we used the TargetScan v.7.1. (http://www.targetscan.org/vert_71/) and miRSystem (http://mirsystem.cgm.ntu.edu.tw).

Those putative miRNA target sites resulting from at least three databases were considered positive. miR-200b target genes selected with these approach were then analyzed using mRNA microarray expression profiles (Affymetrix U133 plus 2.0) to detect gene down and up-regulated genes in TNBC cells as compared to ARRDC3 overexpressing cells. Significance analysis of microarrays (SAM) was conducted to determine differential mRNA expression. The data from Affymetrix microarray expression were analyzed by Mann-Whitney U Test and *p* < 0.05 was considered as significant.

### 2.7. Cell Viability by MTT Assay and Crystal Violet Stain

Cells (3.5 × 10^3^) were seeded in 96-well plates with 100 μL media in triplicate and allowed to adhere overnight. The cells were treated with 5-FU at the concentrations indicated. After the treatment for 24, 48 or 72 h, viability was evaluated using the Kit-8 (Dojundo Molecular Technologies, Rockville, MD, USA) according to the manufacturer’s instructions. Absorption at 450 nm was determined using an iMark Microplate Reader (Bio Rad). IC_50_ values, representing the drug concentration causing 50% growth inhibition, were calculated using https://www.aatbio.com/ tools/ic50-calculator.

For viability staining, cells were transfected with GFP and GFP-ARRDC3. After 24 h, cells were treated with drugs for indicated times. The cells were rinsed with PBS, fixed in methanol and stained with crystal violet. After rinse, plates were air dried overnight. Stained cells were counted and the images were captured by microscope and digital camera (Nikon, Melville, NY, USA)

### 2.8. Cell Cycle Assay

Cells plated on 6-well plate were transfected with miRNAs. After incubation for 48 h, cells were collected by trypsinization, washed with PBS and fixed with 66% ethanol at 4 °C for 1h. Cell were washed with PBS and stained in 200 μL of propidium iodide (PI) and RNase stating solution (PI Flow Cytometry kit for cell cycle analysis, Abcam), followed by incubation at 37 °C for 20 min. For Flow Cytometry, Cells were detached by trypsinization and washed with PBS containing 1% BSA. A total of 1 × 10^6^ cells were incubated with APC-CD44 and PE-CD24 for 30 min on ice. CD24^−^/CD44^+^ populations were detected on a BD FACSAria IIu flow cytometer (BD Bioscience, San Jose, CA, USA). Cell cycle distribution was performed using BD FACSCalibur flow cytometer and analyzed using ModFit LT v3.3 software.

### 2.9. Cell Motility Assay

Cell motility assays were performed by a transwell cell culture chamber of 8 μm pore size (Costar-Falcon, Corning Life Science, Tewksbury, MA, USA) according to the standard procedure. Transwell inserts were coated with collagen I (15 μg/mL) overnight at 4 °C. After washing the inserts with PBS next day, cells were added to the upper chamber of each well. Lysophosphatidic acid (LPA; 100 ng/mL) was added to the lower chambers as a chemoattractant. The chambers were incubated for 2 h at 37 °C with 10% CO_2_. The cells that did not migrate through the pores were mechanically removed by cotton swab. The migrated cells on the lower surface of the membrane were fixed and stained with 0.2% crystal violet and counted. Assays were performed in triplicate and repeated three times.

### 2.10. Colony Formation Assay

MDA-MB-231 cells transfected with control (NC), miR-200b and 5-Fu_miR200b were suspended in the top layer of DMEM (1 mL) containing 0.35% low-melt agarose (ISC Bioexpress, Kaysville, UT, USA) and then the top layer was overlaid on DMEM (2 mL) containing 0.75% agar in six-well plates. The cells were fed twice per week with 0.5 mL DMEM. After 3 weeks, colonies larger than 0.1 mm in diameter were counted per well by using bright-field optics. The images of colonies were assessed by a microscope and digital camera (Nikon). The average number of colonies was obtained from counting triplicate wells.

## 3. Results

### 3.1. ARRDC3 Reverses Chemo-Resistance to DNA Damaging Agents and EMT Phenotypes of Mesenchymal Subtype TNBC Cells

**Figure 1:**
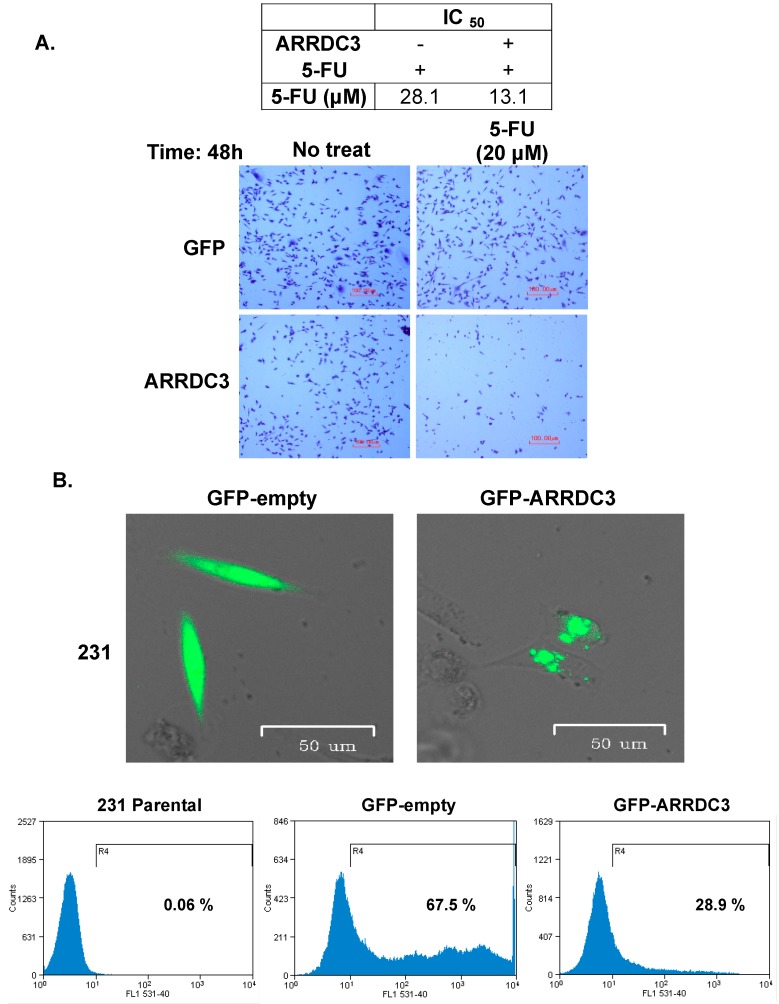

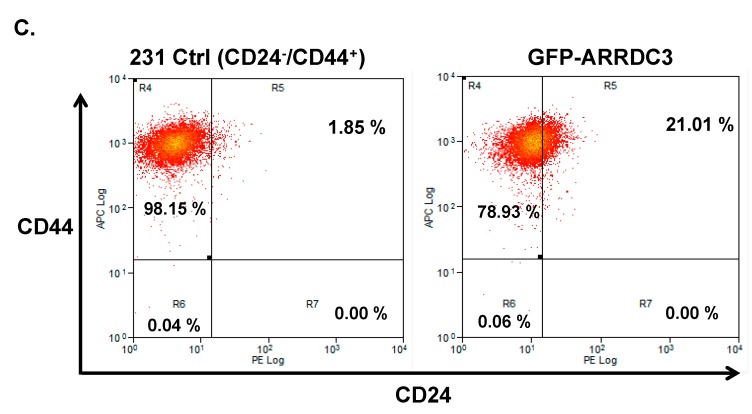
ARRDC3 reverses chemo-resistance to DNA damaging agents in mesenchymal subtype of TNBC cells. (**A**) MDA-MB-231 cells transfected with ARRDC3 cDNA were treated with 5-FU (20 μM) for 48 h (+: presence; −: absence). Cell cytotoxicity was evaluated by crystal violet staining and MTT assay. The values of IC_50_ of 5-FU are shown in tables. (**B**) MDA-MB-231 cells transfected with GFP-empty or GFP-ARRDC3 were imaged by fluoresce microscope and analyzed by flow cytometry. Overlay of DIC images and GFP (green) fluorescence are shown in upper panel. Histograms show percentage of GFP positive cells (lower panel). (**C**) FACS analysis shows population of CD24^−^/CD44^+^ (cancer stem cell marker) in MDA-MB-231cells transfected with GPF and GFP-ARRDC3. Representative all images were selected from three independent experiments.

Our previous studies demonstrated that a metastasis suppressor, ARRDC3 is epigenetically silenced in TNBC cells [8], and that restoring ARRDC3 expression represents an important anti-cancer mechanism of selective inhibitors of nuclear exporters (SINEs) that effectively inhibits TNBC functions in vitro and in vivo [9]. In the current studies, we tested whether the metastasis-suppressing function of ARRDC3 is linked to reduction of chemoresistant nature and epithelial to mesenchymal transition (EMT) phenotypes of TNBC cells. We chose MDA-MB-231 cell line as they represents metastatic mesenchymal subtype of TNBC cell line whose ARRDC3 expression is epigenetically silenced [8] and generally resistant to DNA damaging chemo-agents [18]. We measured and compared cytotoxicity of DNA damaging agent, 5-FU in MDA-MB-231 cells with or without ARRDC3 over-expression (Figure 1A). Overexpression of ARRDC3 in MDA-MB-231 cells sensitizes them to 5-FU as shown in microscopic images and reduction in IC_50_ value to 5-FU (Figure 1A). We then assessed the effects of ARRDC3 expression in phenotypic changes and stemness in MDA-MB-231 cells. As shown in Figure 1B, transient transfection of GFP tagged ARRDC3 yields 28.9% efficiency of GFP-ARRDC3 cDNA expression in MDA-MB-231 cells. GFP-ARRDC3 transfected cells become more round shape (epithelial like) whereas GFP-null vectors transfected cells maintain MDA-MB-231 cells’ typical elongated shape (mesenchymal like) (Figure 1B). We then measured CD24^-^/CD44^+^ population (stemness like) upon transfection of either GFP-null or GFP-ARRDC3 vectors into MDA-MB-231 cells (Figure 1C). Expression of ARRDC3 reduced CD24^−^/CD44^+^ population roughly by 20%, which correspond to transfection efficiency of GFP-ARRDC3 in MDA-MB-231 cells (Figure 1C). These outcomes suggest that ARRDC3 plays a role in reversing epithelial to mesenchymal phenotypes (EMT) phenotypes, stemness and chemo-resistance in TNBC cells.

**Figure 2:**
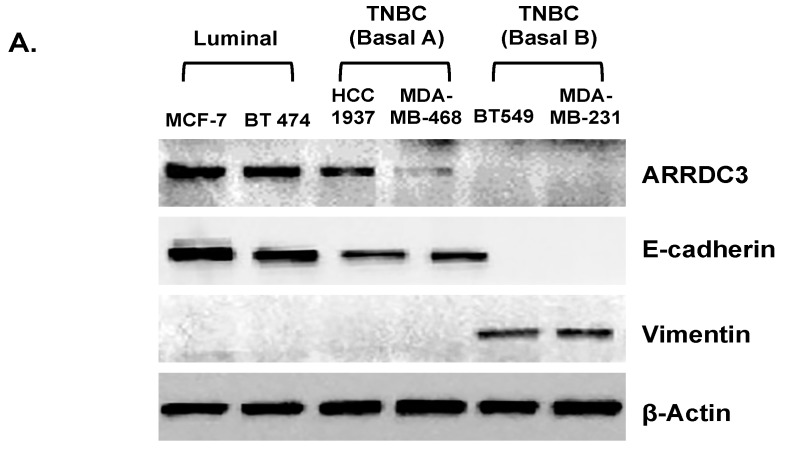

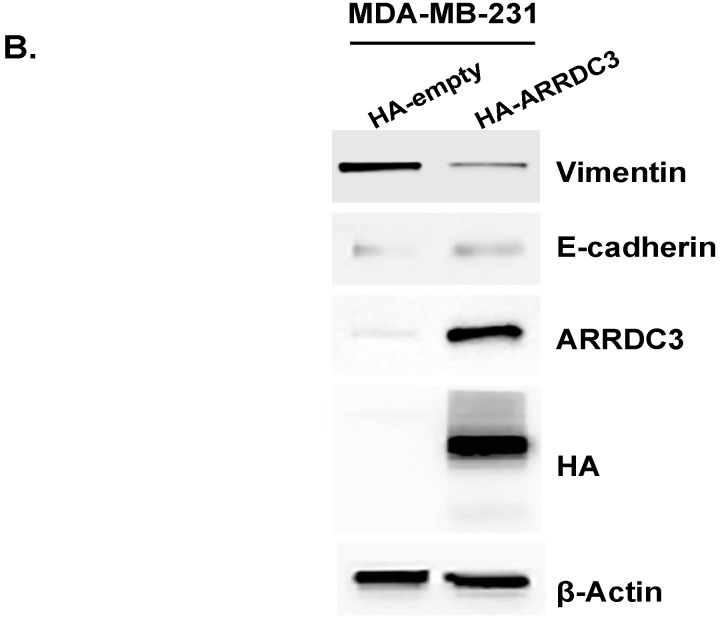
ARRDC3 reverses EMT phenotypes in mesenchymal subtype of TNBC cells. (**A**) Whole cell lysates were prepared from luminal (MCF-7 and BT-474), epithelial subtype of TNBC (basal A; HCC-1937 and MDA-MB-468) and mesenchymal subtype of TNBC (basal B; BT549 and MDA-MB-231) cell lines. Equal amount of lysates were used for western blot analysis by using antibodies against ARRDC3, *E*-cadherin, vimentin and β-actin (loading control). (**B**) The lysates from MDA-MB-231 cells transfected with HA-empty and HA-ARRDC3 vector were analyzed by western blotting with the indicated antibodies. All blot images are representative data of three independent experiments.

To further define relationship between ARRDC3 levels and EMT phenotypes, we selected a panel of breast cancer cell lines with different ARRDC3 levels (Figure 2). As shown in Figure 2A, ARRDC3 levels were very low to undetectable in mesenchymal subtype (Basal B) of TNBC cell lines in comparison to luminal subtype of breast cancer cell lines and epithelial subtype (Basal A) of TNBC cell lines. The loss of ARRDC3 expression in mesenchymal subtype of TNBC cell lines is correlated with loss of E-cadherin (epithelial marker) and presence of vimentin (mesenchymal marker), which are established EMT markers (Figure 2A). The forced expression of ARRDC3 restores the expression of E-cadherin and decreased the levels of vimentin (Figure 2B). The results support the role of ARRDC3 in reversing EMT phenotypes in TNBC cells.

### 3.2. miR-200b-3p Regulates ARRDC3 Expression and Forms a Positive Feedback Loop with ARRDC3 to Reverse EMT Phenotypes and Chemo-Resistance of TNBC Cells

**Figure 3:**
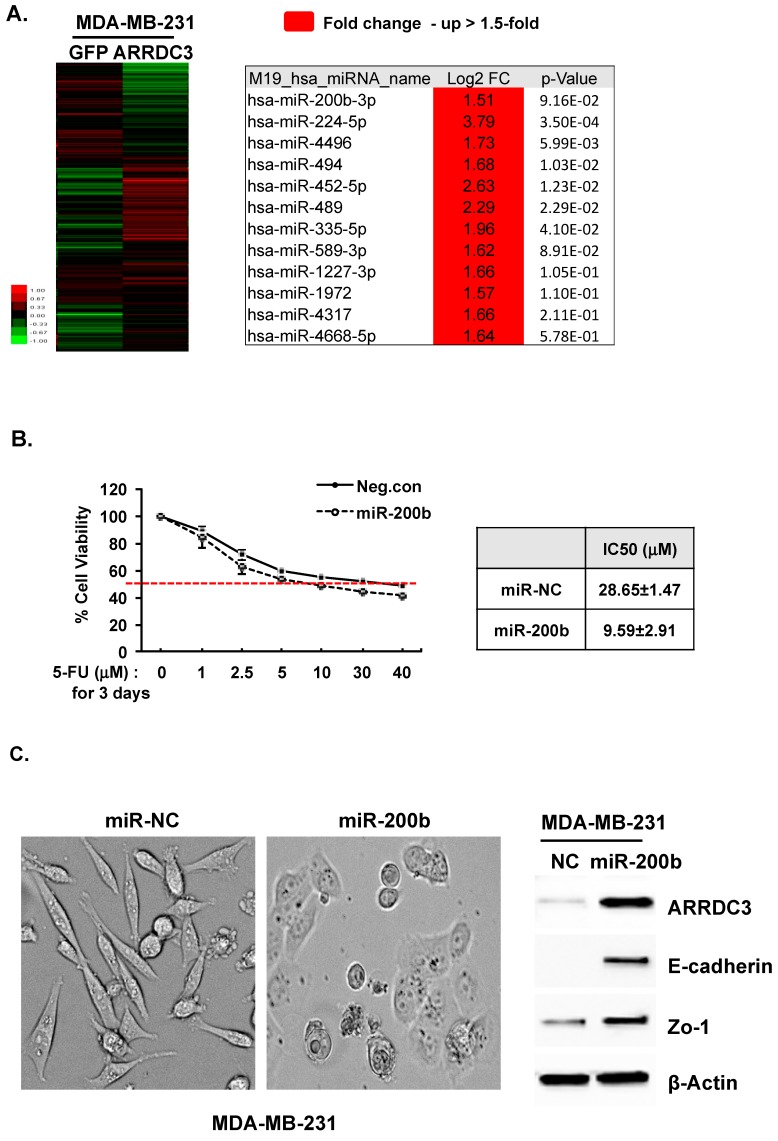

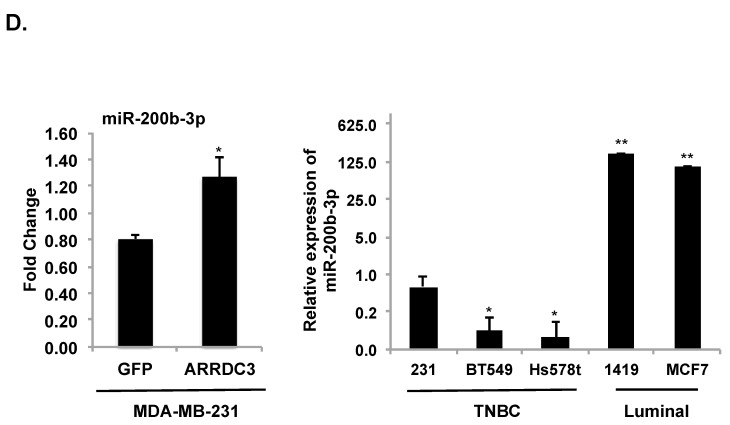
miR-200b-3p regulates ARRDC3 expression and forms a positive feedback loop with ARRDC3 to reverse EMT phenotypes and chemo-resistance of TNBC cells. (**A**) Clustering analysis shows miRNA profile expression between GFP (control) and ARRDC3 overexpressing MDA-MB-231cell (left panel). Signal intensity is values normalized by control gene probes and log_2_-transformed. Right table represents selected miRNAs in red color group of up-regulated miRNAs in ARRDC3-MDA-MB-231 cells (≥ 1.5 fold) as compared to in the GFP control 231 cells. (**B**) MDA-MB-231cell expressing either miR-200b or negative control (miR-NC) was treated with 5-FU for 72 h, and cell viability was measured by MTT assay. (**C**) MDA-MB-231cell were transfected with miR-200b or negative control. The morphological change of cells was imaged by phase contrast microscopy (left panel). The change to EMT phenotype in MDA-MB-231 cells expressing miR-200b was confirmed by Western blot analysis with the indicated antibodies (right panel). β-actin was used as loading control. (**D**) RNAs were isolated from the indicated cells and prepared as described in the Material and Method section. miR-200b expression level was quantitated by qRT-PCR. Column, mean from at least three independent experiments; bars, SD. The statistical analysis was done using Student’s *t* test. *, *p <* 0.05, **, *p <* 0.01.

To identify miRNA(s) that mediate the metastasis suppressing function of ARRDC3, MDA-MB-231 cells that express GFP (transfection control) or ARRDC3 were used for RNA extraction and miRNA array processing. Purified RNAs from each sample were labeled with fluorescent dye and hybridized to the miRNA microarrays overnight. The microarrays were scanned on an Axon Gepenix 4000B scanner, and the data was extracted from images using GenePix software (Figure 3A). We selected a group of mi-RNAs (12 in total) whose roles have been implicated as tumor suppressor, and their levels are up-regulated 1.5 fold or higher by ARRDC3 expression in MDA-MB-231 cells (Figure 3A). We initially screened these mi-RNAs for their ability to reduce the IC_50_ values to 5-FU (in a similar level that ARRDC3 reduces IC_50_ to 5-FU) and found that miR-200b-3p (miR-200b from now on) was most effective among those tumor suppressing miRNAs listed in Figure 3A in sensitizing MDA-MB-231 cells to 5-FU by lowering IC_50_ value from 28.65 to 9.59 μM (Figure 3B). Interestingly, expression of miR-200b also induced the expression of ARRDC3 in MDA-MB-231 cells, suggesting the positive feedback loop between ARRDC3 and miR-200b and reverses EMT phenotypes in MDA-MB-231 cells (Figure 3C). The reversal of EMT phenotype by miR-200b in MDA-MB-231 cells was confirmed as miR-200b expression increased the levels of epithelial markers such as E-cadeherin and ZO-1 (Figure 3C). We confirmed the increase in expression of miR-200b upon ARRDC3 expression in MDA-MB-231 cells by q-RT-PCR (Figure 3D). Comparison of relative miR-200b levels between mesenchymal subtype metastatic TNBC cell lines (MDA-MB-231, BT549, and HS578T) and non-metastatic luminal breast cancer cell lines (HCC1419, MCF7) by qRT-PCR showed that miR-200b expression is down-regulated in TNBC cells (Figure 3D). The outcome once again suggests the correlation between ARRDC3 and miR-200b levels.

### 3.3. 5-FU Incorporated miR-200b (5-FU-miR-200b mimic) Showed Enhanced Therapeutic Efficacy in TNBC Cells

**Figure 4:**
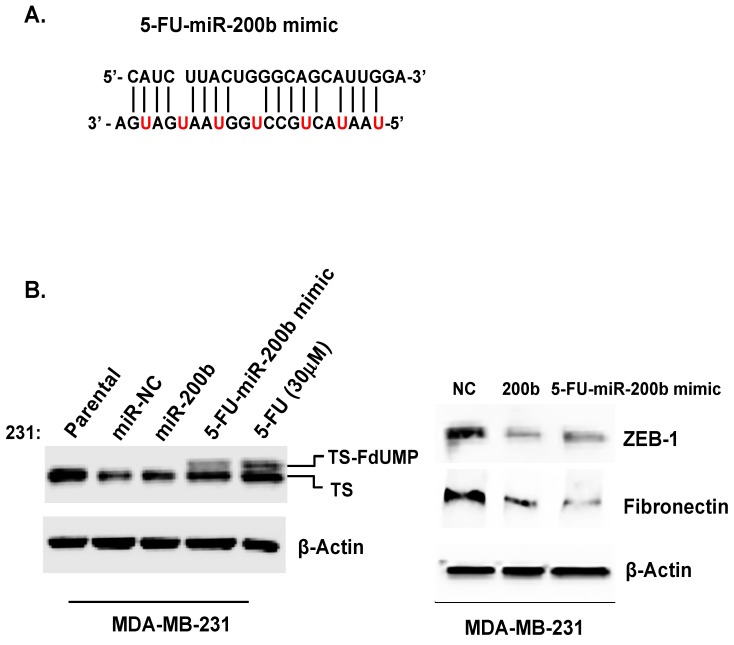

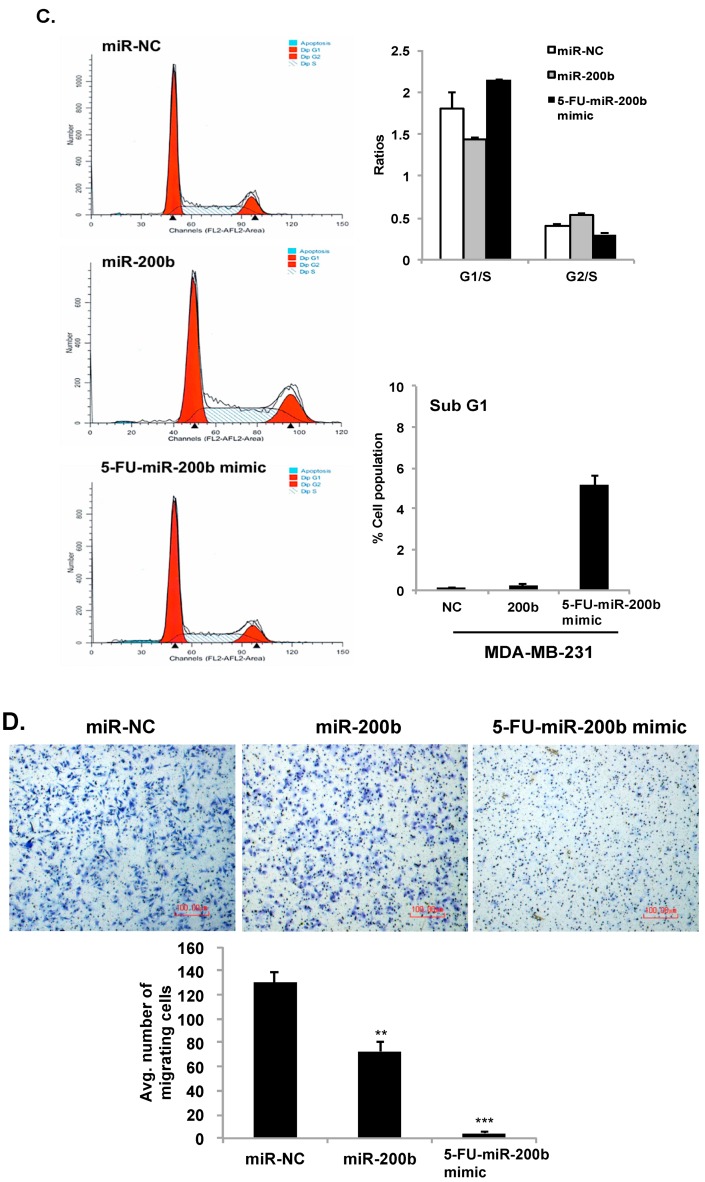

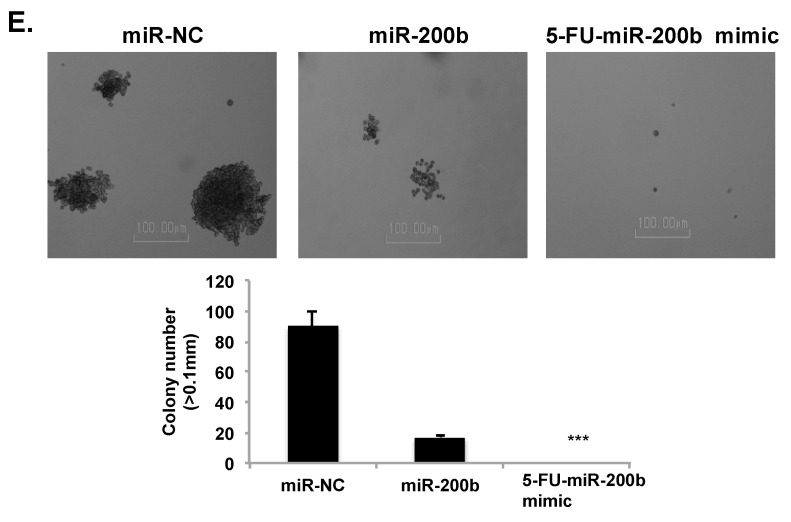
5-FU incorporated miR-200b (5-FU-miR-200b mimic) showed enhanced therapeutic efficacy in TNBC cells. (**A**) Sequence of 5-FU-miR-200b mimic. All uracils in miR-200b were replaced with 5-FU. (**B**) MDA-MB-231 cells were treated with 30 μM 5-FU or transfected with the indicated miRNAs. Cell lysates from each sample were used for western blot analysis. In left panel, 5-FU-miR-200b mimic was shown to maintain functional 5-FU activity by presence of FdUMP-TS complex (the upper shift-band). In right panel, the expression level of ZEB-1 and Fibronectin was detected by Western blot analysis. β-actin levels were used as loading control. (**C**) MDA-MB-231 cells transfected with the indicated miRNAs were stained with propidium iodidie (PI) and the percentage of cell population in each phase was analyzed by flow cytometry. Representative flow cytometry pattern images were selected from three independent experiments (left). The ratio was calculated on the right average values of 3 independent assays (right; bar graph). (**D**) The cell motility inhibitory ability of MDA-MB-231 cells transfected with the 5-FU-miR-200b mimic was measured using a transwell assay. The migration was quantified by counting the cells per square milliliter using bright-field optics. (**E**) MDA-MB-231 cells expressing miR-200b, 5-FU-miR-200b mimic and negative controls were cultured in soft agar containing growth medium after 10 days. Colony formation was captured at 10× or 20× magnification. Only cell colonies >0.1 mm in diameter were counted. Representative images were carried out at least 3 times. All data are presented as the means ± SD. *, *p* < 0.05, **, *p <* 0.01, ***, *p* < 0.001.

Based on result that miR-200b expression sensitizes TNBC cells to 5-FU (Figure 2D), we developed the novel strategy to conjugate the 5-FU into miR-200b (5-FU-miR-200b mimic) into a single agent to maximize the synergistic effects so that low doses of 5-FU-miR-200b mimic can have maximal genotoxic effects on TNBC cells. To generate 5-FU-miR-200b mimic, single-strand RNAs modified with 5-fluorouridine in place of internal U were synthesized (Dharmacon, CO). The 2-ACE protecting groups of RNA oligonucleotides were removed by deprotection reaction according to the manufacturer’s protocol. The equimolar amounts of sense and antisense strand were mixed and annealed to form RNA duplex (Figure 4A). To assess the effects of 5-FU-miR-200b mimic on TNBC cells, MDA-MB-231 cells were transfected with miR-NC (negative control), miR-200b, or 5-FU-miR-200b mimic by using oligofectamine. 5-FU-miR-200b mimic was shown to maintain the miR-200b ability by reducing the expression of known miR-200b target genes such as ZEB-1 and fibronectin and also possess functional 5-FU activity as shown by the detection of the thymidylate synthase FdUMP (TS-FdUMP) complex (Figure 4B).

To assess the effects of miR-200b and 5-FU-miR-200b mimic in cell cycle progression, representative flow cytometry outcome from miR-NC (negative control), miR-200b, or 5-FU-miR-200b mimic transfected cells are shown in Figure 4C. Transfection of miR-200b increases the G2/S ratio whereas transfection of 5-FU-miR-200b mimic significantly increases the sub G1 and G1/S ratio, suggesting different inhibitory mechanisms of cell cycle progression between miR-200b and 5-FU-miR-200b mimic (Figure 4C). Transfection of miR-200b reduced cell motility for 40% and anchorage independent growth for 80% whereas 5-FU-miR-200b mimic essentially blocked both cell motility and anchorage independent growth, suggesting that 5-FU-miR-200b mimic is far more effective in inhibition of TNBC cell functions than miR-200b alone (Figure 4D,E).

### 3.4. Vesicle Free Delivery of 5-FU-miR-200b Mimic

**Figure 5:**
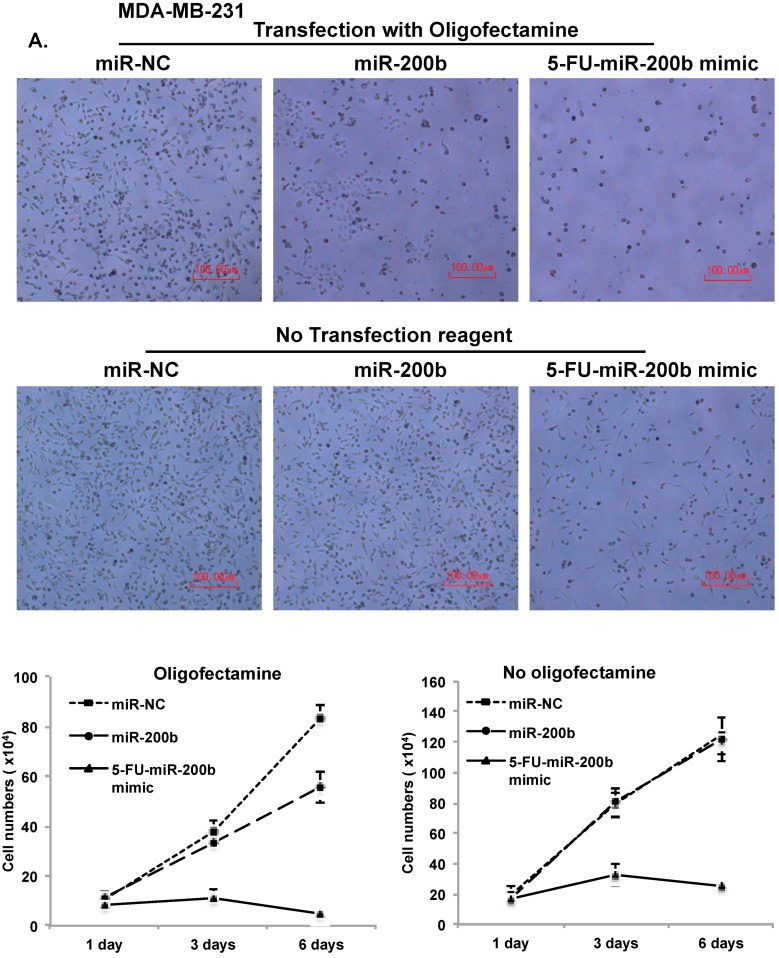

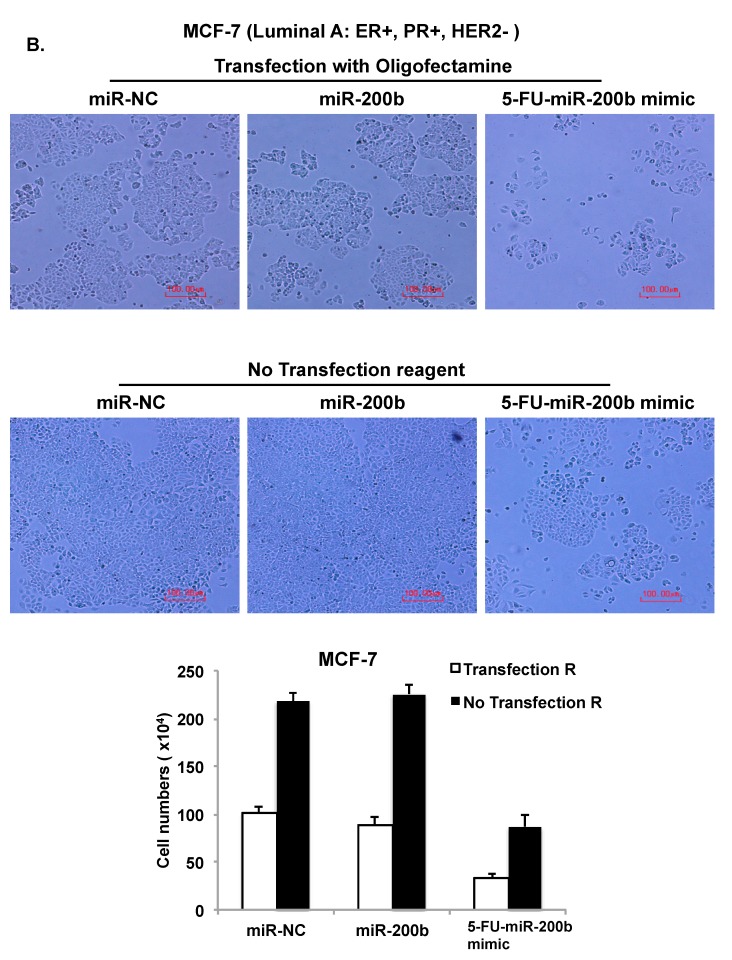

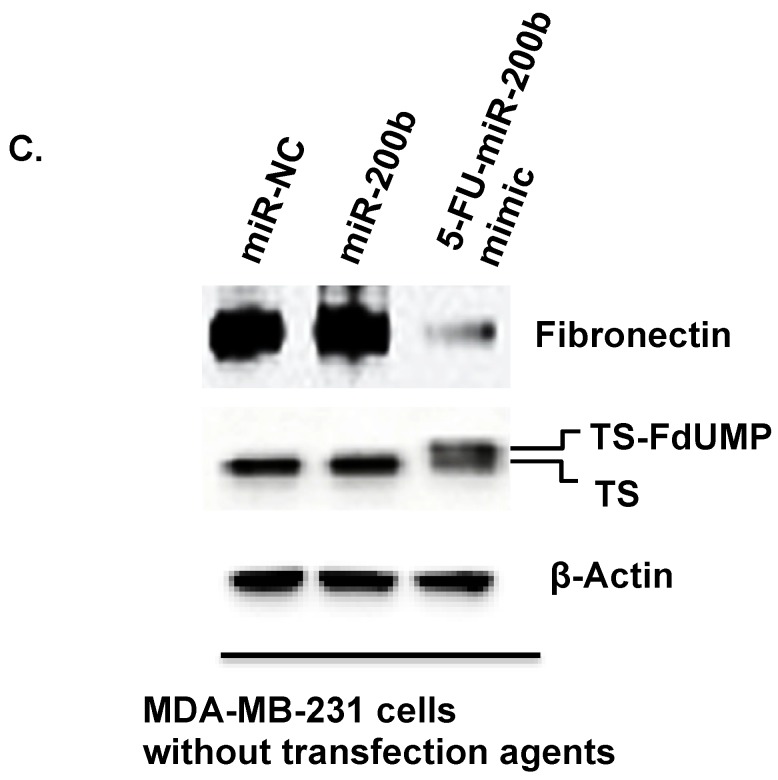
Delivery of 5-FU-miR-200b mimic does not require transfection reagent. miR-negative control (NC), miR-200b and 5-FU-miR-200b mimic were transfected into MDA-MB-231 cells (**A**) or MCF-7 cells (**B**) with or without oligofectamine reagent. Cell proliferation was measured by MTT assay. (**C**) Whole cell lysate from MDA-MB-231 cells transfected with the indicated miRNAs without oligofectamine reagent were analyzed by western blot assay with antibodies against fibronectin, TS-FdUMP and β-actin. Representative images were carried out at least three times.

miRNA delivery into cells generally requires transfection reagents. We found that delivery of 5-FU-miR-200b mimic into cells does not require any transfection agents. MDA-MB-231 cells were transfected with miR-NC (negative control), miR-200b, or 5-FU-miR-200b mimic by using oligofectamine or incubated with these miRNAs without delivery vehicle. The effects of expression of these miRs on TNBC cell proliferation were monitored by MTT assay for 6 days upon transfection or incubation (Figure 5A). While miR-200b reduced the rate of cell proliferation of MDA-MB-231 cells in comparison to miR-NC, 5-FU-miR-200b mimic dramatically reduced the cell growth in a way that cell numbers started to decrease from day 3 (Figure 5A).

Interestingly, 5-FU-miR-200b mimic is still able to block proliferation of MDA-MB-231 cells even without oligofectamine treatment (without delivery vehicle) whereas miR-NC and miR-200b had no effects on proliferation without oligofectamine (Figure 5A). miR-200b has no inhibitory effects on proliferation of luminal subtype of breast cancer cells, MCF7, suggesting that its inhibitory effects can be TNBC specific (Figure 5B). However, 5-FU-miR-200b mimic reduces proliferation of MCF-7 cells with or without oligofectamine (Figure 5B). This outcome indicates that 5-FU-miR-200b mimic has broader range of specificity in terms of its inhibitory functions. Reduction of fibronectin (miR-200b target gene) expression was only observed by 5-FU-miR-200b mimic, but not by miR-NC or miR-200b without oligofectamine (Figure 5C). The results suggest that 5-FU-miR-200b mimic can get into the cells and inhibit TNBC cell proliferation and target gene expression without transfection agents.

## 4. Discussion

Our studies have demonstrated the mechanism by which ARRDC3 reverses EMT phenotypes and chemo-resistance to 5-FU in TNBC cells. miR-200b is an important target gene of ARRDC3 and mediates chemo-sensitizing function of ARRDC3. Based on our outcome that expression of ARRDC3 in TNBC cell is particularly effective in sensitizing TNBC cells to 5-FU over other conventional chemo-drugs, and that miR-200b also sensitize TNBC cells to 5-FU, we anticipate that ARRDC3/miR-200b feedback loop is an important therapeutic target for TNBC. This rationale led to development of novel strategy that combine therapeutic powers of 5-FU and miR-200b into a single compound (5-FU-miR-200b mimic) that can be characterized as a new treatment option for TNBC.

Based on the heterogenous nature of TNBC, it remains to be determined whether therapeutic targeting of ARRDC3/miR-200b pathway is effective in breast cancer in general, or more effective on specific subtypes of TNBC. Our result that ARRDC3 and miR-200b levels are particularly low to absent in the mesenchymal subtype of TNBC cell lines suggest that restoration of ARRDC3 or miR-200b can be more effective in mesenchymal subtype TNBC. Our previous report that IC_50_ of selective inhibitors of nuclear exporter (SINEs) is inversely correlated with the levels of ARRDC3 further supports this hypothesis as restoration of ARRDC3 represents anti-cancer mechanism of SINEs [9]. Our data that miR-200b effectively reduces proliferation of TNBC cell line (MDA-MB-231), but not luminal subtype breast cancer cells (MCF-7) (Figure 5) suggest that targeting ARRDC3/miR-200b pathway can be more effective in TNBC. It is possible that reversal of EMT phenotypes is linked to sensitization of mesenchymal subtype of TNBC cells to DNA damaging agents such as 5-FU whereas non-mesenchymal breast cancer cells are less susceptible by increasing ARRDC3 or miR-200b expression.

We have created a new miRNA mimic by incorporating 5-FU to the therapeutic miRNA, in this case, miR-200b. While 5-FU-miR-200b mimic is technically a single compound, it already possesses the combination therapy component (5-FU as a DNA damaging component and miR-200b as a sensitizing component) in it and this synergistic effect was obvious (Figure 4). Although double stranded 5-FU-miR-200b is stable, it will eventually break down to release 5-FU and other smaller fragments [19]. We have demonstrated that 5-FU released from 5-FU-miR-200b will be able to form the suicide ternary complex 5-FdUMP-TS-THF, which will result a super band shift on the western blot analysis compared to 5-FU control (Figure 4). This clearly demonstrated that 5-FU released from the intact 5-FU-miR-200b will be able to function as a therapeutic 5-FU molecule, and it will be incorporated in to RNA/DNA to influence RNA or DNA synthesis. It is interesting to note that 5-FU-miR-200b mimic seems to possess broader specificity of inhibiting breast cancer subtypes than wild type miR-200b. miR-200b is mainly effective in inhibiting proliferation of TNBC cell line, but not luminal breast cancer cell line whereas 5-FU-miR-200b mimic is effective in inhibiting proliferation of both cell lines (Figure 5). This outcome suggests that 5-FU-miR-200b mimic can be further characterized as pan-breast cancer therapeutic agent including TNBC. The mechanism by which 5-FU-miR-489 mimic enters into the cells without transfection reagent is not defined yet. However, there are multiple evidence supporting that chemical modifications of oligonucleotides confer their ability to get into the cells without transfection agents (called “gymnosis”) [20,21]. While defining the mechanism of cell entry of 5-FU-miR-200b mimic is outside the scope of our current studies, this represents a significant advancement in miRNA based therapeutic development, as delivery technology is a major bottleneck.

## 5. Conclusions

In conclusion, we identified ARRDC3/miR-200b pathway as a key target to reverse EMT phenotypes and chemo-resistance of TNBC cells in the current studies. Our strategy that combines therapeutic powers of 5-FU and miR-200b into a single compound could provide the basis for future trial of 5-FU-miR-200b mimic with other conventional chemotherapy compounds as unique strategy for future breast cancer treatment.
